# Resistance training beyond momentary failure: the effects of past-failure partials on muscle hypertrophy in the gastrocnemius

**DOI:** 10.3389/fpsyg.2025.1494323

**Published:** 2025-02-10

**Authors:** Stian Larsen, Paul Alan Swinton, Nordis Østerås Sandberg, Benjamin Sandvik Kristiansen, Andrea Bao Fredriksen, Hallvard Nygaard Falch, Roland van den Tillaar, Milo Wolf

**Affiliations:** ^1^Department of Sports Science and Physical Education, Nord University, Levanger, Norway; ^2^Department of Sport and Exercise, School of Health Sciences, Robert Gordon University, Aberdeen, United Kingdom; ^3^Department of Exercise Science and Recreation, Applied Muscle Development Lab, CUNY Lehman College, Bronx, NY, United States

**Keywords:** calf raises, proximity-to-failure, muscle thickness, medial gastrocnemius, ultrasound

## Abstract

Muscle hypertrophy is often a desired goal of resistance training, and strategies that extend training beyond momentary failure may enhance muscular adaptations. Thus, the objective of this study was to assess whether performing additional past-failure partial repetitions beyond momentary failure increased muscle hypertrophy. A total of 23 untrained men completed a 10-week within-participant intervention study. This study comprised two weekly resistance training sessions of four sets of standing Smith machine calf raises. One limb was randomly allocated to the control condition performing sets to momentary failure (PLANTAR_MF_), and the other limb was allocated to the test intervention that included additional past-failure partial repetitions in the lengthened position (DORSI_vf_). Muscle thickness of the medial gastrocnemius muscle was measured both pre- and post-intervention via ultrasound. Data were analysed within a Bayesian framework using a mixed-effect model with random effects to account for the within-participant design. The average treatment effect (ATE) was measured to assess any difference in condition and inferences made based on the ATE posterior distribution and associated Bayes Factor (BF). The main findings were that the PLANTAR_MF_ and DORSI_VF_ legs increased medial gastrocnemius hypertrophy by 6.7 and +9.6%, respectively. The results identified an ATE favouring the inclusion of additional partial repetitions (0.62 [95%CrI: 0.21–1.0 mm; *p*(>0) = 0.998]) with ‘strong’ evidence (BF = 13.3) supporting *a priori* hypothesis. Therefore, when the goal is to train for maximum gastrocnemius hypertrophy over a relatively short time period, we suggest performing sets beyond momentary failure as a likely superior option.

## Introduction

Resistance training is often used to increase skeletal muscle mass and strength. In resistance training, the repetitions performed lie on a continuum. Typically, a set—the most extreme termination point of a cluster of uninterrupted repetitions—is considered to be a momentary failure (Refalo et al., 2024). Momentary failure has been defined as the set ending when an athlete reaches a point where, despite attempting to do so, they cannot complete the concentric portion of a repetition without any deviation from the prescribed form of the exercise (Steele et al., 2017). Volitional failure, another set of failures refers to the point at which an individual perceives they have reached the set termination criteria (Refalo et al., 2022). Despite differences observed in set failure definitions, when sufficient proximity-to-failure is achieved, progressive recruitment of high-threshold motor units occurs; it exposes type II myofibers to mechanical tension (Refalo et al., 2022). Although the underlying mechanisms of hypertrophy are poorly understood, mechanical tension currently appears as the most likely and influential stimulus responsible for inducing muscle hypertrophy (Wackerhage et al., 2019).

Proximity-to-failure has been observed to acutely influence several physiological responses during resistance training, such as muscular damage and neuromuscular fatigue (Pareja-Blanco et al., 2017). Such physiological responses following resistance training may reduce the muscle’s capability to contract in subsequent training sessions (Refalo et al., 2022), thereby potentially limiting mechanical tension and the resulting muscle hypertrophy. Recently, Robinson et al. (2023) conducted an exploratory meta-regression, and observed that training to, or close to, momentary failure meaningfully improved hypertrophic outcomes compared to training further from momentary failure. The joint angles at which momentary failure occurs, however, may vary across exercises as the internal moment arm and length-tension relationships likely influence the maximal possible muscle torque generation at a joint (Kipp et al., 2022; Ottinger et al., 2023). In addition, the chosen range of motion (ROM) in the resistance exercise may influence the hypertrophy stimulus due to several factors, such as the length-tension relationship, internal moment arm, and activation of the muscle (Ottinger et al., 2023; Kassiano et al., 2023). It potentially influences the mechanical tension experienced by the target musculature during resistance training. For example, several studies have reported that a full ROM enhances hypertrophy compared to partial ROM (Bloomquist et al., 2013; Kubo et al., 2019). This occurs, especially when the partial ROM is performed at shorter muscle lengths in relation to the average muscle length achieved during full ROM training (Wolf et al., 2023). Though some studies have observed greater muscular adaptations for training with full ROM compared to partial ROM at shorter muscle lengths, a recent meta-analysis by Wolf et al. (2023) revealed favourable effects of partial ROM performed at longer muscle lengths. For example, Pedrosa et al. (2022) observed larger distal quadriceps femoris hypertrophy in a cohort of untrained women when performing the leg extension exercise in a lengthened partial ROM (100–65° of knee flexion) compared to full ROM (100–30°) and shortened partial ROM (65–30°) conditions. Therefore, though the mechanisms remain largely unclear, it has been hypothesised that resistance training at longer-muscle lengths is beneficial when the desired outcome is hypertrophy, potentially due to the muscle reaching the plateau or descending limb increasing mechanical tension experienced at the sarcomere level (Ottinger et al., 2023). Additionally, it has been proposed that a partial ROM may enhance muscle deoxygenation and blood lactate accumulation (Goto et al., 2019), which could mechanistically explain divergent physiological adaptations to resistance training (Wolf et al., 2023). Neither of these explanations currently has a sufficient evidentiary basis to justify a high degree of confidence, highlighting the need for further research.

Kassiano et al. (2023) compared muscle thickness changes in young women performing calf raises with a full ROM (−25° dorsiflexion to +25° plantarflexion), a lengthened partial ROM (−25 to 0°), or a shortened partial ROM (0 to +25°). The investigators observed larger increases in medial gastrocnemius muscle thickness for the lengthened partial ROM compared to the other conditions. However, the full ROM group reached momentary failure at approximately 0° to +25° plantar flexion. Mechanistically, this indicates that the sets were terminated when the muscle was at a weaker force production position, as the gastrocnemius is thought to produce lower torque at plantarflexion angles (10–30° plantarflexion) compared to dorsiflexion angles (−20° to 10° dorsiflexion) with an extended knee (Maganaris, 2003). However, the same study also reported larger internal gastrocnemius moment arms at the plantarflexion angles (Maganaris, 2003). These findings indicate that if the set is terminated at momentary failure, the gastrocnemius operates at the ascending limb and therefore could be in a weaker force position, potentially limiting the mechanical tension achieved by the muscle. Past-failure partials may involve performing additional partial repetitions in the lengthened ROM after reaching momentary failure, where the muscle can no longer complete a full ROM repetition. This approach allows for continued mechanical loading in the portion of the movement where the muscle can generate force, thereby potentially increasing both the total volume load and the mechanical tension experienced by the target musculature. Thus, continuing the set past the inability to achieve peak plantarflexion at a given load, combining full repetitions with subsequent partial repetitions could constitute a better strategy to increase both the intra-set volume and the mechanical tension experienced by the gastrocnemius muscle. As a result, this could plausibly increase hypertrophic adaptations experienced by the gastrocnemius muscle.

Importantly, several studies have compared different proximity-to-failure conditions on muscle hypertrophy (Robinson et al., 2023). To our knowledge, however, no studies have compared sets terminated at momentary failure with sets terminated beyond momentary failure comprising full then initial partial repetitions (hereafter referred to as “past-failure partials”) on muscle hypertrophy. As a result, this study aimed to compare standing calf raises with full ROM in a Smith machine to volitional failure in peak dorsiflexion (combining full repetitions with partial repetitions) with momentary failure in peak plantarflexion on medial gastrocnemius hypertrophy among untrained men. It was hypothesised that terminating the set around peak dorsiflexion would result in favourable hypertrophic outcomes of the medial gastrocnemius compared to terminating the set at peak plantarflexion.

## Materials and methods

### Experimental approach

The current study followed a within-participant repeated-measures design. Each participant had their right and left limb randomly allocated^1^ to one of the two failure conditions: momentary failure reached in peak plantar flexion ROM (PLANTAR_MF_), or volitional failure reached in peak dorsiflexion ROM (DORSI_VF_). Randomization and allocation procedures were performed prior to beginning the study and concealed from the investigators. All participants performed standing calf raises in a Smith machine (Smith Machine N, GymSport AS, Trondheim, Norway) with an individualised ROM (see Supplementary File 1) based on the individual ankle dorsiflexion and plantarflexion flexibility. This flexibility was assessed both before and after the training intervention. The study lasted 12 weeks, with two outcome measurement sessions performed both at the beginning of week one and at the end of week 12. One familiarisation session was performed to introduce the participants to the respective techniques. In this session, the participants worked up to their 10–20 RM on both legs unilaterally. Thereafter, all participants trained in unilateral standing Smith machine calf raises for 10 weeks, with a training frequency of two times per week, except for the first week and week 11, where only one resistance training session was performed. This led to a total of 18 training sessions. Between weeks 2 and 5 the participants trained each leg with three sets each workout with their 10–20 repetition maximum, whereas in weeks 6–11 all participants performed four sets during each workout. During the pre- and post-training intervention measurement sessions, medial gastrocnemius muscle thickness was assessed via b-mode ultrasonography (see Supplementary File 1). Data collection was performed between January 2024 and March 2024 in Levanger, Norway.

### Risk of bias

This study aimed to follow the Standards Method for Assessment of Resistance Training in Longitudinal Design (SMART-LD) checklist for the items that could reduce the chance for potential biases (Schoenfeld et al., 2023) (see Supplementary File 1). This resulted in a final grading of 19 out of 20 points (>80%), which is categorised as good quality (Schoenfeld et al., 2023). Additionally, a study protocol with a pre-registered hypothesis was registered prior to data collection on the Open Science Framework.^2^ Importantly, this study originally aimed to investigate the effect of momentary failure in peak dorsiflexion vs. peak plantarflexion. However, many of the participants were unable to perform all sets due to momentary failure in peak dorsiflexion even with strong verbal encouragement. Therefore, momentary failure in peak dorsiflexion was changed to volitional failure around peak dorsiflexion after agreement within the research group.

### Study participants

**Figure 1:**
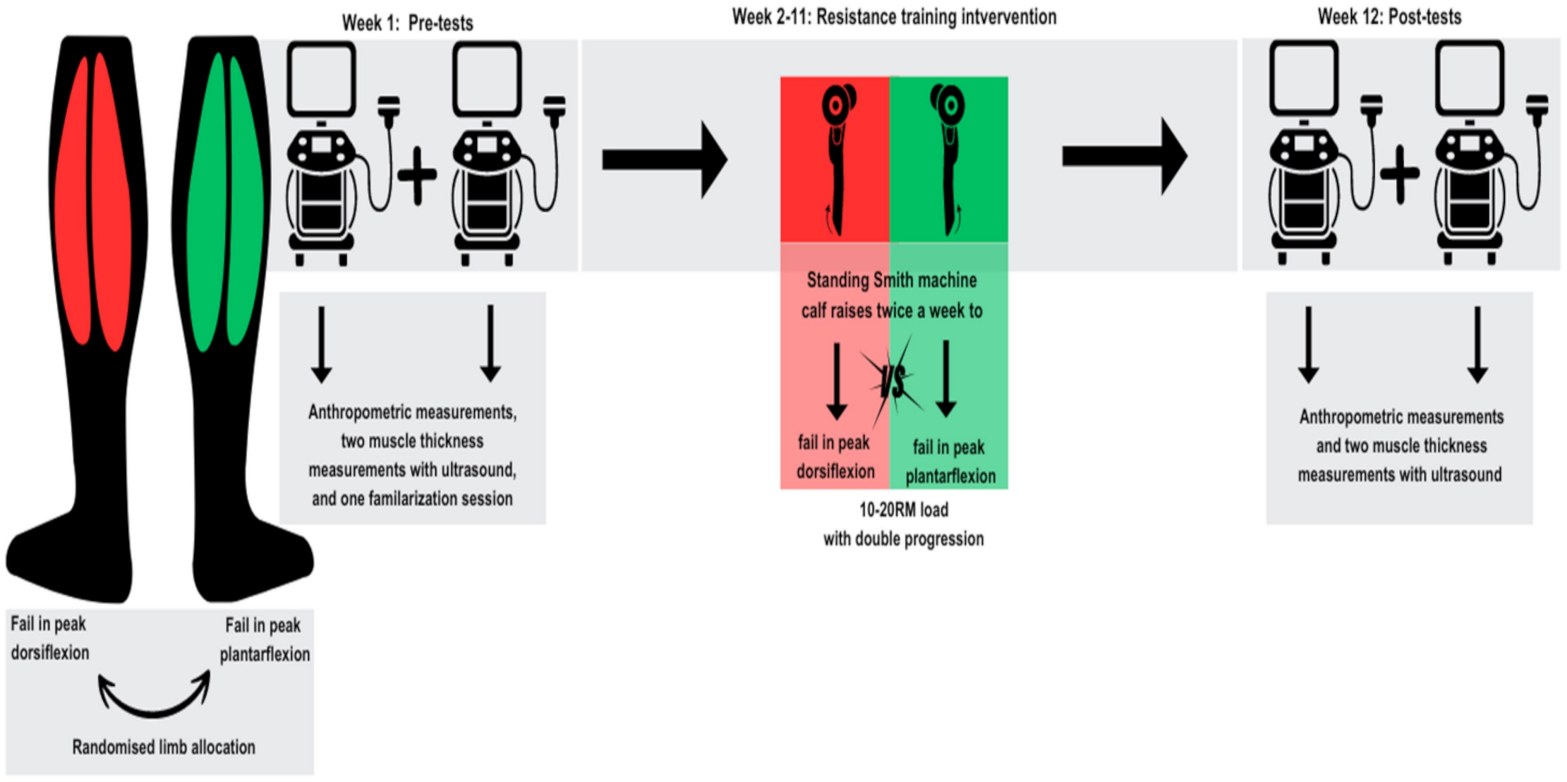
A schematic overview of the study design. RM: repetition-maximum. Graphics inspired by Refalo et al. (2024).

**Figure 2:**
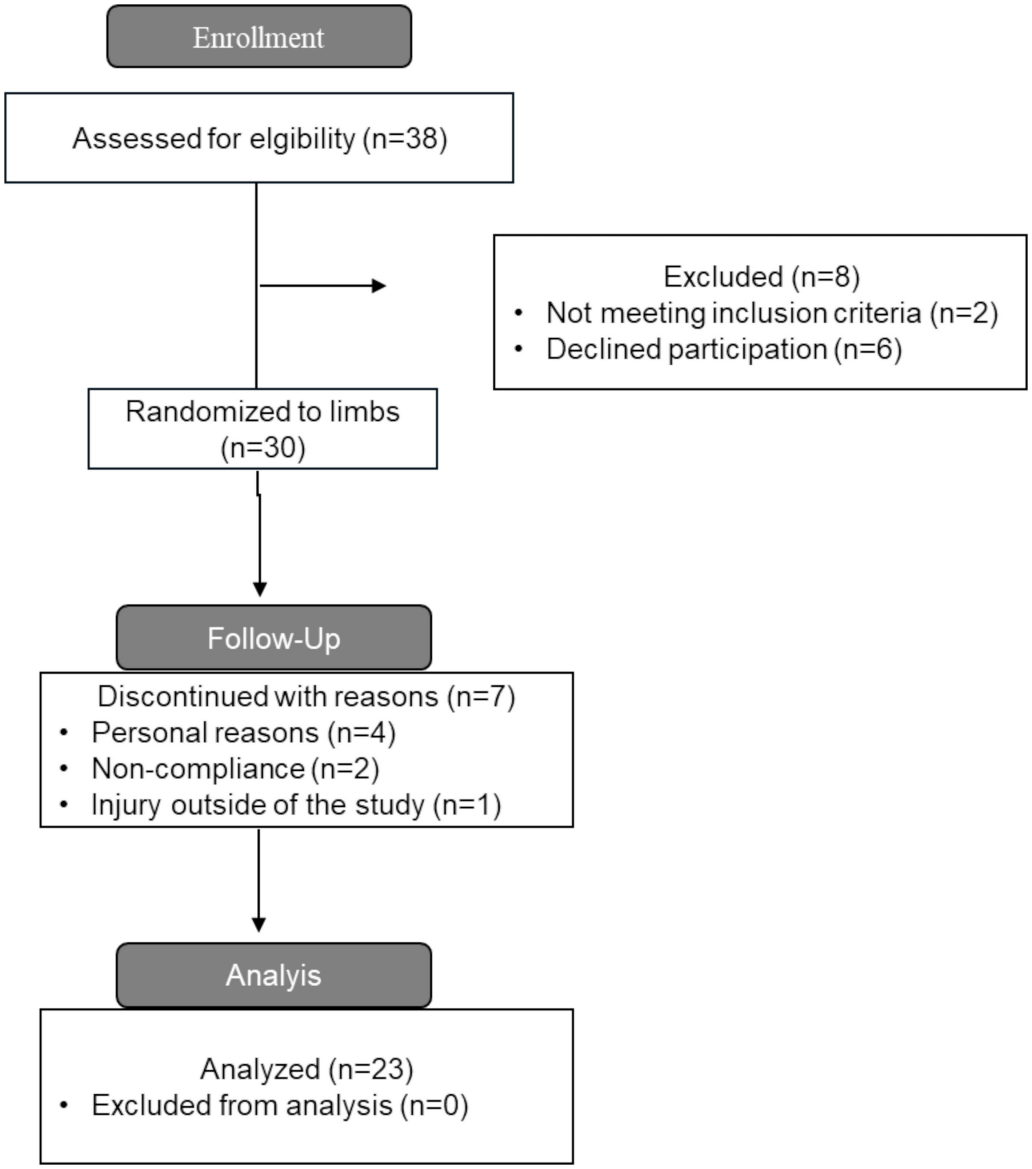
Consort diagram showing the data collection process.

A total of 30 healthy, untrained adult men aged 18–50 years were recruited for this study. A wide age range was selected to ensure an adequate number of recruited participants. Inclusion criteria were: (1) age 18–50 years, (2) no resistance training experience, which was defined as having performed less than one session a week in the past 6 months, (3) no previous self-reported use of illegal muscle-enhancing agents such as anabolic steroids, (4) and no cardiorespiratory or musculoskeletal disorders that could limit maximal performance on the training sessions. To be included in this study, the participants had to complete at least 16 training sessions, which corresponded to attendance of 85% (Figure 1). This criterion resulted in a final sample of 23 participants who were included in the analysis (body mass: 84.6 ± 12.8 kg, age: 31.8 ± 6.1 years, height: 180.3 ± 5.7 cm; Figure 2). The participants were instructed to maintain their daily nutritional schedules during the training intervention; however, all participants were recommended to consume a daily protein intake of at least 1.6 g of protein for each kg of body mass (Morton et al., 2018). A detailed oral description of study procedures was provided before commencing the study. All participants signed a written informed consent form before the start of the study. The project was approved by the Norwegian Agency for Shared Services in Education and Research (Approval number: 125855) and adhered to the latest revision of the Helsinki Declaration. Additionally, ethical approval was obtained from the Regional Committee for Medical and Health Research Ethics, which deemed the study exempt from ethical presentation (Approval number: 696927).

### Sample size rationale

While *a priori* power analyses are typically conducted to determine a sample size that ensures a “desirable” lever of statistical power for detecting the smallest effect size of interest, the actual limitation for recruitment is often based on resource constraints (Lakens, 2022). Therefore, we aimed to recruit as many participants as our resources permitted to maximise statistical power. To further bolster power and increase the precision of estimations, we employed a within-participant design with two baseline tests at two posttests (Swinton, 2024). This resulted in the recruitment of 30 volunteers.

### Procedures

The same procedures at two pre-intervention and two post-intervention tests for medial gastrocnemius muscle thickness measurements were conducted via b-mode ultrasonography (Echo Wave 2 Software, Telemed, Lativa). The medial gastrocnemius was selected for investigation due to its demonstrated excellent reliability measures in previous studies utilizing ultrasonography (Kassiano et al., 2023; Nunes et al., 2020). A 9-MHz scanning frequency with a 60-mm probe size, and Chemolan transmission gel (Chemodis, DA, Alkmaar, The Netherlands) were used for all measurements. The participants were asked to rest in a supine position for 10 min before measurements began. After these measurements, the participant switched to a prone position, and muscle thickness measurements of the medial gastrocnemius were performed. Images were obtained longitudinally (see Supplementary File 1) for both legs, with the probe positioned at the most prominent and thickest site of the leg from a posteroanterior view (Kassiano et al., 2023). The same two assessors (S.L and B.S.K) performed all measurement procedures. One assessor handled the probe, while the other assessor captured the scan. Once satisfactory quality of the muscle thickness measurements was ascertained, three ultrasound images were obtained from both limbs during both pre- and post-intervention tests, totalling six images from both limbs during both pre- and post-intervention testing (12 images in total). Averages of three measurements taken within a single testing session were used to perform subsequent analyses. A pen was used to draw reference lines on the medial gastrocnemius. Pictures of the reference lines for all participants were taken to enable accurate pre- and post-intervention test values. All reference line pictures and muscle thickness measurements were saved and stored in a password-secured external flash drive, which only the two assessors had access to. The first post-intervention test was performed at least 120 h after the last training session, whereas the second post-intervention test was performed approximately 144–168 h after the last training session. The participants were instructed to avoid food and caffeine consumption for 2 and 8 h prior to the measurement sessions, respectively. Importantly, the reliability of measurements was evaluated between the two pre-intervention tests, and the two post-intervention tests using the intraclass correlation coefficient (ICC), coefficient of variation (CV), and typical error (TE). For the DORSI_VF_ leg, an ICC, CV, and TE of 0.98, 1.91%, and 0.39 mm were achieved between pre-intervention tests, while for the PLANTAR_MF_ leg, the values were 0.97, 1.82%, and 0.37 mm, respectively. Reliability measures between post-interventions yielded an ICC, CV, and TE of 0.98, 1.39%, and 0.31 mm for the DORSI_VF_ leg, and 0.99, 0.80%, and 0.18 mm for the PLANTAR_MF_ leg. Furthermore, all participants were asked two questions on the last post-intervention test: (1) Would you prefer training the standing calf raise on your own to failure in the bottom position if you knew this would increase muscle growth compared to the condition of reaching failure in the top position?; (2) If yes, how much more relative muscle growth compared to the standing calf raise failed at the top position would be needed for choosing the condition reaching failure in the bottom position?

Resistance training was performed twice per week with a minimum of 48 h rest between sessions for 10 weeks. The calf raise was performed unilaterally in a Smith machine (Gymsport, Tiller, Norway) with the foot standing on a step with anti-skid tape added to increase friction (see Supplementary File 1). All repetitions were executed with a full ROM repetition range between 10 and 20 repetitions until momentary failure. The PLANTAR_MF_ terminated the set when reaching momentary failure, defined as being unable to reach the same barbell height on the failed repetition as the first repetition. The DORSI_VF_ continued with partial repetitions after reaching momentary failure until the participant was unable to ascend over the point of peak dorsiflexion (meaning not able to plantarflex the talocrural joint from the deepest position) or upon reaching volitional failure around peak dorsiflexion. Importantly, joint angle ROM was individualised to achieve both peak dorsiflexion and plantarflexion, thereby achieving the greatest lengthened and shortened position achievable with an extended knee for each subject. Barbell load was increased in 0.25–0.50 kg increments when the participant was able to perform 20^+^ repetitions with full ROM on the first set. To measure and count full ROM repetitions in the Smith machine calf raise, the participants were asked to plantarflex the ankle joint as much as possible on the first repetition while the personal trainer measured the peak barbell height during the first repetition of the calf raise and marked this height with a finger, which was kept in place throughout the set. Thus, peak plantarflexion ROM was individualised on each set to the highest plantarflexion achievable. The PLANTAR_MF_ set was terminated when the barbell did not reach the same barbell height as the first repetition, whereas partial repetitions were counted for the DORSI_VF_. In addition, volume load was calculated as sets x repetitions x load.

During familiarisation, all participants gradually increased their workload to one set at approximately 20 repetitions maximum. During the first training week, all participants performed one session with three sets, each with momentary failure. From weeks 2–5, all participants completed two sessions with three sets each, totalling six weekly sets. In weeks 6–9, all participants completed two sessions with four sets each, with a total of eight weekly sets. In week 10, only one session with four sets was performed. This was done to ensure that muscle edema and swelling did not confound the muscle thickness measurements as the DORSI_VF_ performed more repetitions than the PLANTAR_MF_ leg.

Rest periods were set at approximately 30 s between legs and more than 120 s between sets (new set = when both legs were trained). Limb training order was changed each week, meaning that in weeks 1, 3, 5, 7, and 9, participants started exercising with the DORSI_VF_ limb, whereas, in weeks 2, 4, 6, 8, and 10, the participants started exercising with the PLANTAR_MF_ limb. All participants were instructed to perform the eccentric component with around 2-s duration, whereas the concentric was instructed to be performed with maximal intended velocity.

### Statistical analysis

Statistical analysis was performed in R (version 4.4.0) using a Bayesian framework. The Bayesian approach enabled us to use formal inclusion of information regarding likely differences between interventions based on knowledge from former studies with the use of informative priors. This enabled the use of inferences to be based on intuitive probabilities and, therefore, assessed the strength of evidence in support of the existence or nonexistence of an intervention effect (Schad et al., 2022). The main analysis was conducted using a linear mixed-effects model with random effects to account for both the within-participant design and repeated measures (Magezi, 2015). The effect of set termination criteria on muscle thickness was quantified through the average treatment effect (ATE). Inferences were based on: (1) a summary of the posterior distribution of the ATE estimate; and (2) the Bayes factor (BF) to quantify the strength of evidence for either a non-zero ATE (alternative hypothesis H_1_) or zero ATE (null hypothesis H_0_). Furthermore, standard qualitative labels that express the strength of evidence for the different hypotheses were used (Lee and Wagenmakers, 2014). The R wrapper package brms was used for all analyses, interfacing with Stan to perform the sampling (Bürkner, 2017), and BFs were estimated via the bridge sampling algorithm (Gronau et al., 2017). We adopted a full Bayesian workflow for the analyses, which was comprised of: (1) the use of informative priors derived from meta-analyses within the field (Swinton et al., 2022); (2) an assessment of the appropriateness of priors using prior predictive checks; (3) running models and evaluating the stability of estimates through repeated iterations with the data; (4) determining the appropriateness of posterior distributions using posterior predictive checks and sensitivity analyses with non-informative priors; (5) conducting simulation-based calibration of BFs (Schad et al., 2022). To enhance transparency, accuracy, and replication for the analyses, we used the WAMBS checklist (When to Worry and How to Avoid Misuse of Bayesian Statistics) (Depaoli and Van de Schoot, 2017). Workflow summaries are reported in Supplementary File 3.

## Results

Mean attendance among the participants who finished the intervention was 17.3 of 18 (96.10%) resistance training sessions. A total of 23 out of 30 participants finished the intervention. Four participants dropped out due to personal reasons, two participants dropped out due to non-compliance, and one participant dropped out due to injury outside the study.

### Muscle morphology

**Figure 3:**
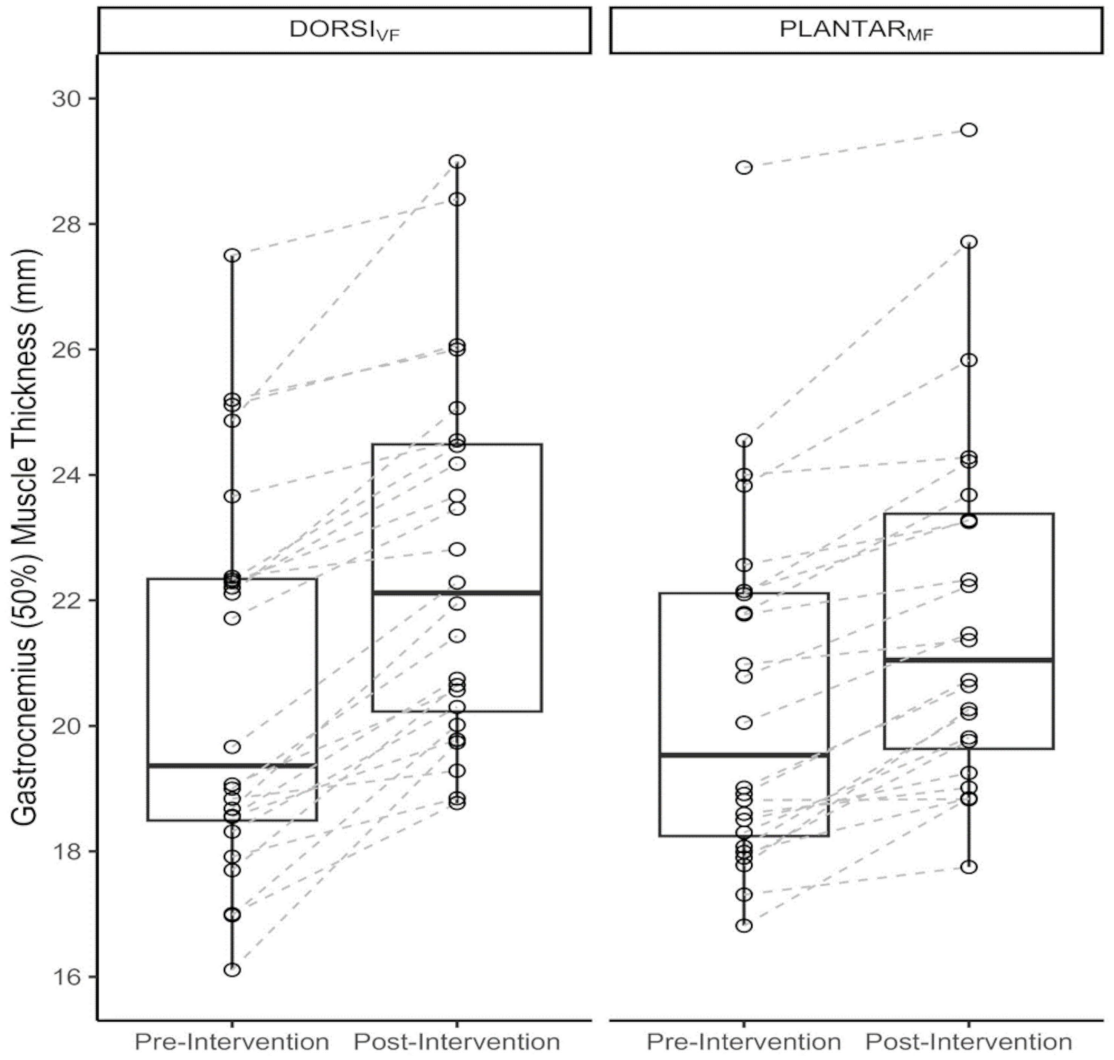
Box plots with individual pre- and post-intervention muscle thickness values for the medial gastrocnemius. Individual data points are calculated from the average of the two measurement sessions at that time period and separated by the two conditions (DORSI_VF_: beyond momentary failure and including partial repetitions in the dorsiflexed position; and PLANTAR_MF_: to momentary failure in the plantarflexed position).

A descriptive summary of the training-induced increases in muscle hypertrophy is presented in Table 1, and individual participant data are illustrated in Figure 3. An increase in muscle thickness was observed for the gastrocnemius in both the DORSI_VF_ and PLANTAR_MF_ conditions. The linear mixed model provided ‘strong’ evidence in support of the *a priori* hypothesis that greater increases in muscle hypertrophy would occur with DORSI_VF_ compared to PLANTAR_MF_ (0.62 [95%CrI: 0.21–1.0 mm; *p*(>0) = 0.998; BF = 13.3]).

**Table 1:** Descriptive summaries of data (mean ± SD).

Outcome	Baseline DORSI_vf_	Post-test DORSI_vf_	Δ%	Baseline PLANTAR_MF_	Post-test PLANTAR_MF_	Δ%
Medial gastrocnemius (mm)	20.6 ± 3.1	22.5 ± 2.9	9.6	20.4 ± 2.9	21.8 ± 3.0	6.7

### Questions

Eighteen participants (78.30%) answered yes that they would prefer DORSI_VF_ if this would lead to more muscle growth than training to PLANTAR_MF_. On question two, participants answered that DORSI_VF_ would have to be 32.2 ± 20.7% (range: 5–80%) more effective in increasing muscle growth to prefer training with this condition relative to PLANTAR_MF_.

### Volume load

**Figure 4:**
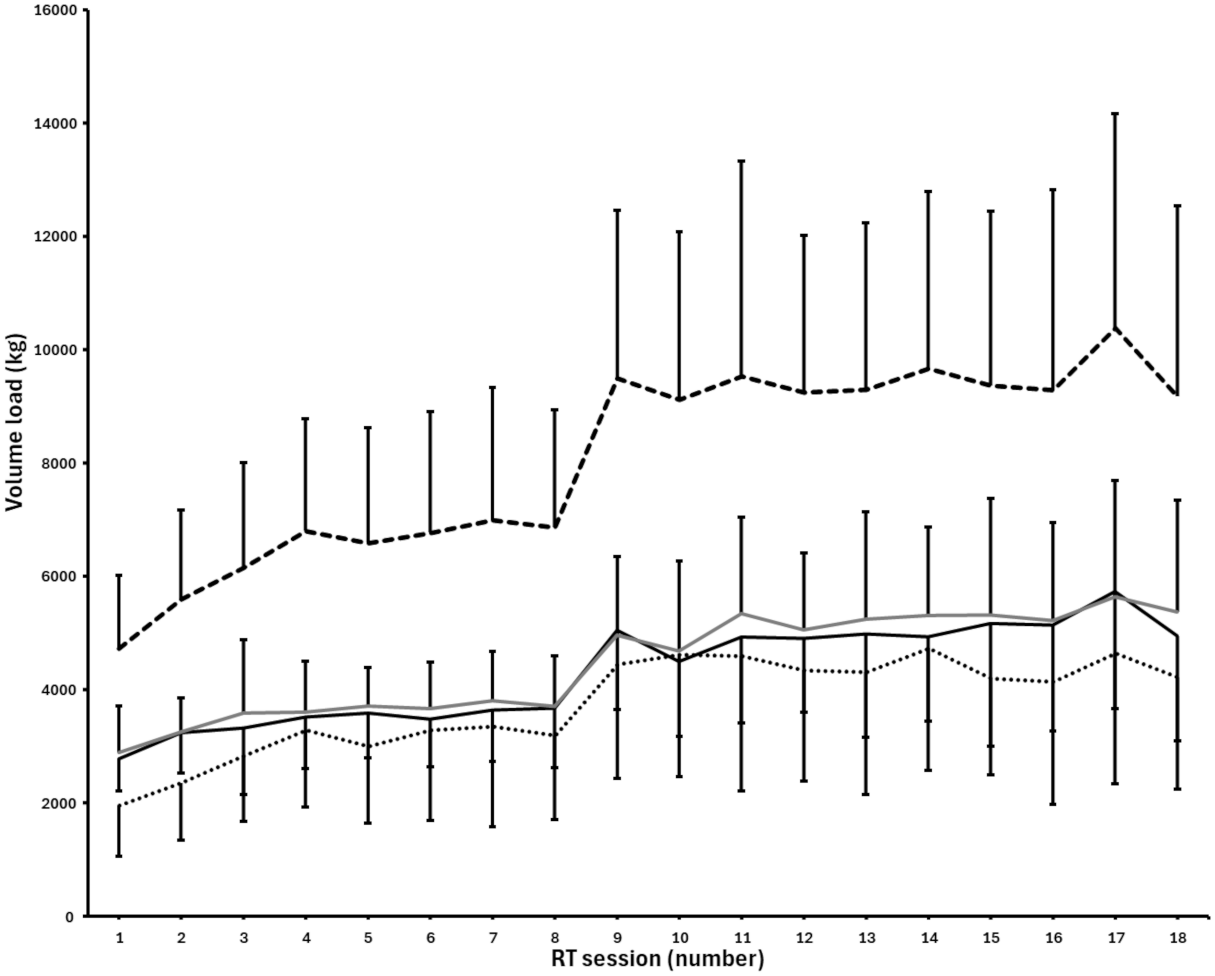
Mean volume load lifted each resistance training session for the DORSI_VF_ and PLANTAR_MF_ group in unilateral Smith machine calf raises. Black solid line = volume load for full repetitions for DORSI_VF_. Black dotted line = volume load for partial repetitions for DORSI_VF_. Black dashed line = total volume load for all repetitions for DORSI_VF_. Grey solid line = total volume load for full repetitions for PLANTAR_MF_.

The total volume load increased from 4,742 ± 1,283 kg and 2,892 ± 821 kg in the first training session to 9,170 ± 3,370 kg and 5,377 ± 1,960 kg in the last session for the DORSI_VF_ limb and PLANTAR_MF_ limb, respectively (Figure 4). The partial volume load performed after reaching momentarily failure was 87.2 ± 7.8% of the full repetitions volume load for the DORSI_VF_ leg with a range from 70 to 103%. The total volume load lifted during the training intervention was 145,141 kg, and 80,417 kg for the DORSI_VF_ limb and PLANTAR_MF_ limb, respectively.

## Discussion

In support of our *a priori* hypothesis, the key finding of the present study was that implementing past-failure partials was superior to training to only momentary failure in inducing hypertrophy of the gastrocnemius following 10 weeks of training among untrained men. Both training interventions resulted in average increases in hypertrophy (DORSI_VF_: +9.6% vs. PLANTAR_MF_: +6.7%). Moreover, the participants could perform an average of 87.20% more volume load for the DORSI_VF_ leg after reaching momentarily full range of motion failure, and the PLANTAR_MF_ trained with only 55.4% of the total volume load compared to the DORSI_VF_ leg.

Notably, we observed an absolute and relative difference of 2.90 and 43.30%, respectively, in favour of the DORSI_VF_ condition. In absolute terms, the model estimate was that the DORSI_VF_ condition could be expected to result in an additional 0.62 mm (95%CrI, 0.21–1.0 mm) increase in muscle thickness compared to PLANTAR_MF_. Within the Bayesian framework, this result represents ‘strong’ evidence in support of our pre-registered hypothesis. The superior hypertrophic outcome for the DORSI_VF_ leg can be related to several factors. First, the PLANTAR_MF_ leg, on average, trained with only approximately 55.40% of the intra-session volume load compared to the DORSI_VF_ leg. This suggests that the DORSI_VF_ leg trained beyond what is often considered to be momentarily failure in the rest of the literature (Refalo et al., 2024; Steele et al., 2017; Robinson et al., 2023). The greater intra-set volume load observed for the DORSI_VF_ was achievable since the set was not terminated when participants were no longer capable of ascending to the same barbell height as the first repetition, as was the case in the PLANTAR_MF_ condition. The fact that participants could perform an average of 87.20% more volume load by including partial repetitions after reaching what is traditionally considered a momentary failure in that foot may indicate that the gastrocnemius and/or soleus operate on the ascending part of their length-tension relationship during and around peak plantar flexion in Smith machine calf raises, as previously documented by Maganaris (2003). Thus, our findings suggest that terminating sets in the joint angle where the gastrocnemius is in a weaker force production position (possibly due to active insufficiency) may reduce hypertrophic outcomes, as participants achieved an average of 87.2% more volume load when performing partial repetitions after reaching what is often considered momentarily failure in the literature. Nevertheless, our results should be generalised to other muscle groups/exercises with caution, as other muscles may have different strength curves, and different exercises may operate on different parts of the length-tension curve (Ottinger et al., 2023), resulting in varying amounts of partial repetitions being achievable after a full range of motion failure. Importantly, our study design did not investigate the mechanisms of why performing past-failure partials contributed to greater hypertrophy. Alternatively, our findings may partly be explained by the fact that the DORSI_VF_ leg was trained at longer muscle lengths, on average, as partial repetitions after full range of motion failure were performed in the lengthened portion of the calf raise exercise (Wolf et al., 2023). This finding was corroborated by Kassiano et al. (2023) who also observed greater gastrocnemius hypertrophy when performing lengthened partials vs. a full ROM. While the mechanisms underlying the potential benefit of lengthened partials remain poorly understood, previous investigations have reported greater increases in resting levels of IGF-1 (McMahon et al., 2014), muscle deoxygenation, increases in blood lactate, and surface electromyography amplitude (Goto et al., 2019) when compared to a full range of motion. Another potential explanation for superior growth was implementing past-failure partials after reaching; this is considered a momentary failure in the literature, thereby providing similar explanations as to why drop sets may be a time-efficient strategy when training for muscle growth (Coleman et al., 2022; Sødal et al., 2023).

To our knowledge, this is the first study to examine the effects of performing repetitions beyond momentary failure in Smith machine calf raises and combining full and partial repetitions on muscle hypertrophy in the medial gastrocnemius. Therefore, it is not possible to draw direct comparisons between the results of the present study in the DORSI_VF_ leg to hypertrophy observed in the gastrocnemius in previous studies. However, the changes in medial gastrocnemius muscle thickness (DORSI_VF_: +9.6%, PLANTAR_MF_: +6.7) were comparable with the values reported in Kassiano et al. (2023) (full ROM: +6.7%, partial initial ROM: 15.2%) for the medial gastrocnemius. Participants in the initial partial ROM/lengthened partial group in Kassiano et al. (2023) study increased muscle thickness from 1.58 ± 0.26 cm to 1.82 ± 0.27 cm after 8 weeks of resistance training. This resulted in approximately 8.5% absolute and 126.9% greater absolute and relative medial gastrocnemius growth for the lengthened partial group compared to the full ROM group. To compare, our DORSI_VF_ group increased from 20.6 ± 3.1 mm to 22.5 ± 2.9 mm resulting in around 2.9% absolute and 43.3% greater relative growth in favour of the DORSI_VF_ leg compared to the PLANTAR_MF_ leg. Importantly, since the participants who trained calf raises through a full ROM and terminated the sets at short muscle lengths in both Kassiano et al. (2023) and in the present study at average increased medial gastrocnemius hypertrophy with 6.70 and 6.70% (equally), we speculate that performing initial partial repetitions in calf raises may be more effective than past-failure partials. However, this speculation requires further investigation, and a direct comparison of past-failure partials and lengthened partials for muscle hypertrophy is warranted.

We observed that the participants frequently reported more discomfort when training due to volitional failure around peak dorsiflexion during the training intervention. This raises the question of whether the training provided beyond full ROM momentary failure and thus experiencing additional discomfort are worth an expected average treatment effect of 0.62 mm in muscle hypertrophy. When the participants were asked if they would prefer training the standing calf raise on their own to failure in the bottom position if they knew this would increase muscle growth compared to the condition of reaching failure in the top position, 18 out of 23 participants answered yes. The group also reported that they would prefer implementing past-failure partials after reaching momentary failure if the training provided a relative growth benefit of 32.2 ± 20.7% (range: 5–80%). Using average values, the preferential threshold fell below the relative growth benefit of 43.30% that we observed when performing past-failure partials after reaching momentary failure. In addition to the five participants who would not prefer implementing past-failure partials into the set even if this approach resulted in greater muscle growth, 6 of the 23 participants (26.1%) highlighted the need for a growth benefit larger than 43.3% to create a preference for training past-failure partials instead of regular sets. This resulted in 12 out of 23 participants (52.2%) preferring to perform past-failure partials after reaching momentary failure based on the relative growth benefit we observed in this study. Notably, the questionnaire was only responded to by participants who completed the intervention, as seven participants discontinued participation during the intervention phase, adding more complexity when trying to draw conclusions. Nonetheless, we hypothesise that the additional muscle hypertrophy attained through training past-failure partials with Smith machine calf raises may justify the additional perceived discomfort for around 50% of individuals. Importantly, this speculation should be considered cautiously and further investigated as we did not use a validated tool to assess perceived discomfort or preferential thresholds, and participants reported a wide range on the question set.

### Strengths and limitations of this study, and further research

This training intervention aimed to be both internally and ecologically valid. We conducted two pre- and two post-intervention tests, pre-registered the study’s methods, and hypotheses, and used the SMART-LD checklist to reduce potential biases. Additionally, the statistical analysis was performed blinded, with condition allocation concealed. These practices increase the robustness of our conclusions and limit the potential for bias. The most prominent limitation of the current study in terms of ecological validity and generalizability to more trained populations was that it measured only muscle hypertrophy among untrained men due to resource constraints. As this to our knowledge is the first study on the effects of implementing past-failure partials on muscle growth, we underline the need for conducting more studies in the future to enhance the body of knowledge. First, future studies should attempt to replicate the findings among women and more highly trained participants. Additionally, future studies should compare muscle hypertrophy from implementing past-failure partials vs. traditional sets on other muscle groups, as our findings are perhaps not generalizable to other muscle groups due to differences in length-tension relationships and resistance curves of the exercises employed. Also, a study comparing past-failure partials to initial partial ROM sets is warranted. Similarly, as we observed increased perceived discomfort among our participants, future studies should compare perceived discomfort with a validated tool between a set implementing past-failure partials after reaching momentary failure vs. a traditional set terminated at momentary failure. Another limitation of the study is the absence of biochemical markers, which could have provided additional insights into the mechanical and metabolic changes induced by the training intervention. Although we used a within-subject design, no non-training control group was included which may limit the ability to quantify the degree of measurement error over time (Hammert et al., 2024). Finally, future studies should compare the efficacy of one past-failure partial set with a drop set, as a time-efficient modality, and 2–3 traditional sets, to compare the effectiveness between these training modalities.

### Practical applications

To increase medial gastrocnemius hypertrophy, past-failure partials may be incorporated into a set after reaching full ROM failure among untrained individuals, However, this approach may increase the perceived discomfort. Thus, trainers and practitioners should consider individual preferences when implementing past-failure partials into a training programme.

## Conclusion

In summary, we demonstrated that medial gastrocnemius muscle hypertrophy was greater when Smith machine calf raises were performed to DORSI_VF_ compared to PLANTAR_MF_. Thus, when the goal is to increase medial gastrocnemius hypertrophy among untrained men, we suggest performing Smith machine calf raises to volitional failure in peak dorsiflexion.

## Data Availability

The raw data supporting the conclusions of this article will be made available by the authors without undue reservation.

## Footnotes

^1^Using www.randomizer.org.

^2^OSF: https://osf.io/kyha9.
