# Supplementary material for: Resistance training beyond momentary failure: the effects of past-failure partials on muscle hypertrophy in the gastrocnemius
Source: Front Psychol. 2025 Feb 10;16:1494323. doi: 10.3389/fpsyg.2025.1494323 (PMC11847862; doi:10.3389/fpsyg.2025.1494323)
Supplement: Supplementary file 1 [file Supplementary_file_1.pdf]

Supplementary file 1.

The data collection this study is based on has three different research questions, resulting in three different articles. All three research questions are pre-registered: see https://osf.io/gvzfa/ and https://osf.io/5689h for the pre-registrations of the two other studies.

1.1. Standard method for assessment of resistance training in longitudinal design (SMART-LD) check-list for the items possible to try to reduce the chance for potential biases (Schoenfeld et al., 2023).

	Item	Description
	**General**	
1.a)	The purpose of the study was clearly stated	Yes. The aim of this study was to compare standing calf raises with full ROM in a Smith machine to volitional failure in peak dorsiflexion. vs. momentary failure in peak plantarflexion on gastrocnemius hypertrophy among untrained men.
2.a)	The study was pre-registered prior to data collection for the outcomes of interest.	Yes. This study was pre-registered prior to study on the Open Science Framework. DOI 10.17605/OSF.IO/BY8KE
	**Participant**	
3.a)	Sample size provided adequate statistical power or was appropriately justified.	Yes. We recruited as many participants as possible based on our resources. In addition, we used a within-participant design to bolster the power.
4.a)	Inclusion/exclusion criteria were adequately identified.	Yes. Inclusion criteria were 1) age-range between 18-50 years, 2) untrained in resistance training, which was defined as conducting less than one session a week in the last six months, 3) no previous self-reported use of illegal muscle-enhancing agents such as anabolic steroids, 4) no cardiorespiratory or musculoskeletal disorders that could limit maximal performance on the training sessions.
5.a)	Subject characteristics were clearly described.	Yes. Mean and standard deviations for age, body mass, and height.
6.a)	Reasons for dropouts were adequately reported.	Yes. Consort flow chart is provided where all dropouts are reported.
7.a)	The study must report attendance and the mean participation must be ≥90% of the total number of sessions provided in the program.	Subjects who failed to participate at least 85% of training sessions (<17 sessions) were excluded from the analyses. Mean attendance was 96.1%.
	**Program**	
8.a)	Training program was written with sufficient detail so that the procedures can be replicated.	Yes. Se resistance training program in table 1.2.
9.a)	Participants were randomly allocated between groups.	Yes. The left and right foot were randomly assigned with www.randomizer.org to one out of the two limbs before the start of the study.
10.a)	Randomization was concealed from investigators and participants.	Yes. Randomization of limbs performed with randomized.org and concealed from investigators and participants prior to study.

11.a)	Training was directly supervised.	Yes. Personal trainers supervised all RT sessions with a 1:1, 1:2, 2:3 supervisor: subject ratio.

	**Outcomes**	
12.a)	Assessments were written with sufficient detail so that the procedures can be replicated.	Yes. See methods.
13.a)	The primary outcome(s) were blinded to investigators.	No. Since the same investigators supervised all RT sessions this was not possible. However, all statistics was performed by a statistician blinded for group allocation.
14.a)	Assessments employed validated methods for the purpose of the primary outcomes.	Yes. Muscle thickness measurement was taken by ultrasound imaging (Echo Wave 2 Software; Telemed, Latvia) with a 60-mm probe size and 9 MHz scanning frequency, and Chemolan transmission gel (Chemodis, DA Alkmaar, The Netherlands).
15.a)	Proper preparation was employed for assessment methods where applicable.	Yes. Participant preparation: The subjects were instructed to not engage in any type of physical activity or training 96 hours before the pre- and post-test. Also, the subjects were instructed to not consume caffeine 8 hours or food 2 hours before the pre- and post-tests. Also, post-test was taken with at least 168 hours after last RT session.
16.a)	Test-retest reliability measures were reported for assessments of the primary outcome(s) where applicable.	Yes. ICC values between the two pre-tests and post-tests are reported in the methods section.
	**Statistics**	
17.a)	Statistical analyses were written with sufficient detail so that the procedures can be replicated.	Yes. See statistics.
18.a)	Appropriate statistical tests were used for outcomes where applicable.	Yes. See statistics
19.a)	Pre- and post-study means, and variability and/or confidence intervals, were reported for all conditions in primary outcomes.	Yes. See statistics and results.
20.a)	Exact values were provided for reported statistics.	Yes. See results.
	**Final grading: 19/20 points.**	

1.2.Resistance training protocol 1 and 2 used in the study.

**RT1**	**Exercise**	**Sets**	**Repetitions**	**Intensity**	**Rest pause**	**Progression method**	**Note**
A1	Biceps curl peak shoulder extension	3 (week 1-5)<br>4 (week 5-10)	10-20	Momentary failure	30 seconds to a2	Double progression (10-20)	Start with a1 week 1, 3, 5, 7, and 9
A2	Biceps curl neutral shoulder extension	3 (week 1-5)<br>4 (week 5-10)	10-20	Momentary failure	120 seconds to a1	Double progression (10-20)	Start with a2 week 2, 4, 6, and 8
B1	Leg extension 40 degrees hip flexion	3 (week 1-5)<br>4 (week 5-10)	10-20	Momentary failure	30 seconds to b2	Double progression (10-20)	Start with b1 week 1, 3, 5, 7, and 9
B2	Leg extension 90 degrees hip flexion	3 (week 1-5)<br>4 (week 5-10)	10-20	Momentary failure	120 seconds to b1	Double progression (10-20)	Start with b2 week 2, 4, 6, and 8
C1	Standing calf raise failure peak plantarflexion	3 (week 1-5)<br>4 (week 5-10)	10-20	Momentary failure in peak plantarflexion	30 seconds to c2	Double progression (10-20)	Start with c1 week 1, 3, 5, 7, and 9
C2	Standing calf raise failure peak dorsiflexion	3 (week 1-5)<br>4 (week 5-10)	10-20	Volitional failure around peak dorsiflexion	120 seconds to c1	Double progression (10-20)	Start with c2 week 2, 4, 6, and 8
**RT2**	**Exercise**	**Sets**	**Repetitions**	**Intensity**	**Rest pause**	**Progression method**	**Note**
A1	Standing calf raise failure peak plantarflexion	3 (week 1-5)<br>4 (week 5-10)	10-20	Momentary failure in peak plantarflexion	30 seconds to a2	Double progression (10-20)	Start with a1 week 1, 3, 5, 7, and 9
A2	Standing calf raise failure peak dorsiflexion	3 (week 1-5)<br>4 (week 5-10)	10-20	Volitional failure around peak dorsiflexion	120 seconds to a1	Double progression (10-20)	Start with a2 week 2, 4, 6, and 8
B1	Leg extension 40 degrees hip flexion	3 (week 1-5)<br>4 (week 5-10)	10-20	Momentary failure	30 seconds to b2	Double progression (10-20)	Start with b1 week 1, 3, 5, 7, and 9
B2	Leg extension 90 degrees hip flexion	3 (week 1-5)<br>4 (week 5-10)	10-20	Momentary failure	120 seconds to b1	Double progression (10-20)	Start with b2 week 2, 4, 6, and 8
C1	Biceps curl peak shoulder extension	3 (week 1-5)<br>4 (week 5-10)	10-20	Momentary failure	30 seconds to c2	Double progression (10-20)	Start with c1 week 1, 3, 5, 7, and 9
C2	Biceps curl neutral shoulder extension	3 (week 1-5)<br>4 (week 5-10)	10-20	Momentary failure	120 seconds to c1	Double progression (10-20)	Start with c2 week 2, 4, 6, and 8

1.3.Alternative RT session.

Exercise	Sets	Repetitions	Repetitions in reserve	Rest pause	Progression method
Leg curl	3	8-12	1-2	120 seconds	Double progression (8-12)
Bench press	3	8-12	1-2	120 seconds	Double progression (8-12)
Shoulder press	3	8-12	1-2	120 seconds	Double progression (8-12)
Cable pullover	3	8-12	1-2	120 seconds	Double progression (8-12)
Triceps extensions	3	8-12	1-2	120 seconds	Double progression (8-12)

The subjects were allowed to train the alernative RT session twice a week since none of these RT exercises trains the biceps brachii, quadriceps femoris, or gastrocnemius effectively. Variations of the resistance exercises were permitted. Variations allowed for the leg curl included: sitting and lying leg curls. Variations allowed for the bench press included: barbell, dumbbell, and machine chest press, with options for incline, horizontal, and decline. Variations allowed for the shoulder press included: barbell, dumbbell, and machine shoulder press, also with options for incline, horizontal, and decline. Variations allowed for the pullover included: rope, handle, and dumbbell pullovers. Variations allowed for the triceps extension included: neutral and overhead triceps extensions. Additionally, subjects was allowed to train the rectus abdomins and erector spinae with RT exercises such as rope chrunches and back hyperextensions.

1.4. Visual demonstration of RT technique.

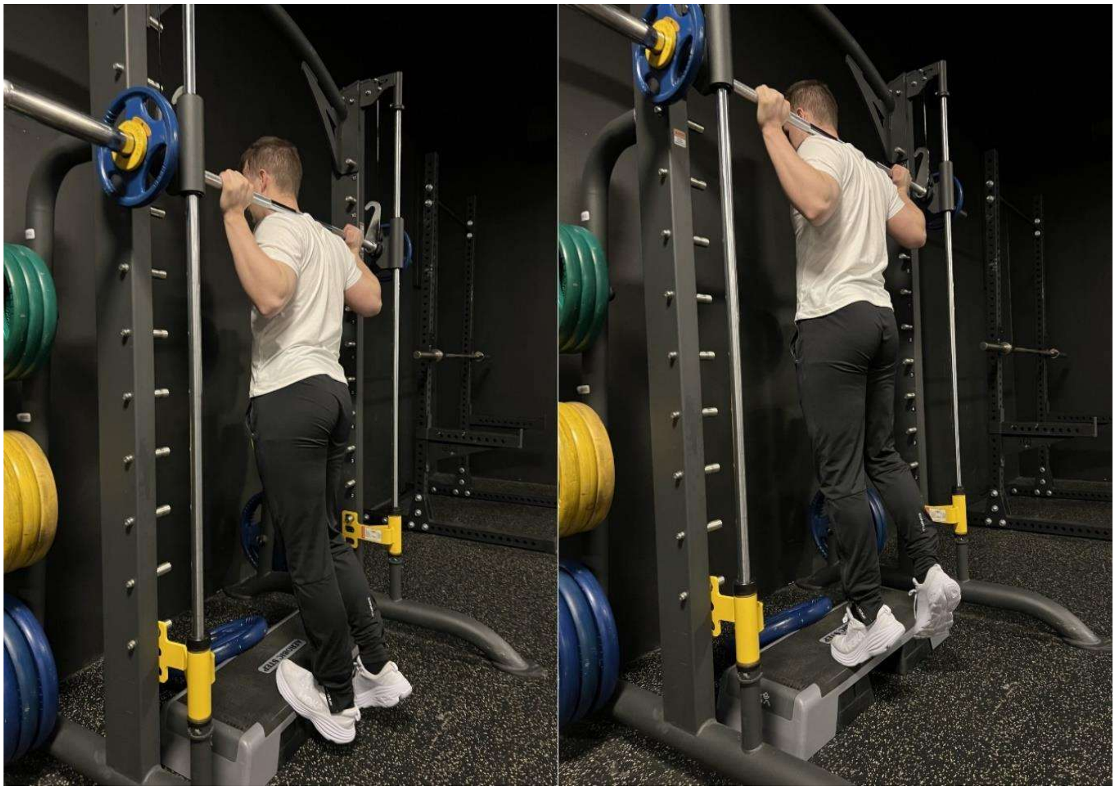

Left shows an example of individualized peak dorsiflexion. Right shows an example of individualized peak plantarflexion. All subjects were instructed to start the first repetition at each set from peak dorsiflexion and ascend to peak plantarflexion. Thereafter, the research assistant put a finger at the peak barbell height reached at the first repetition at each set. Full repetitions were counted each time the barbell touched the finger. When subjects were unable to ascend to the same barbell height as achieved at the first repetition, the set was stopped at the $\text{PLANTAR}_{\text{MF}}$ foot, whereas partial repetitions were counted on the DORSI_{VF} foot until participants reached volitional failure around peak dorsiflexion.

1.5. Example of ultrasound images.

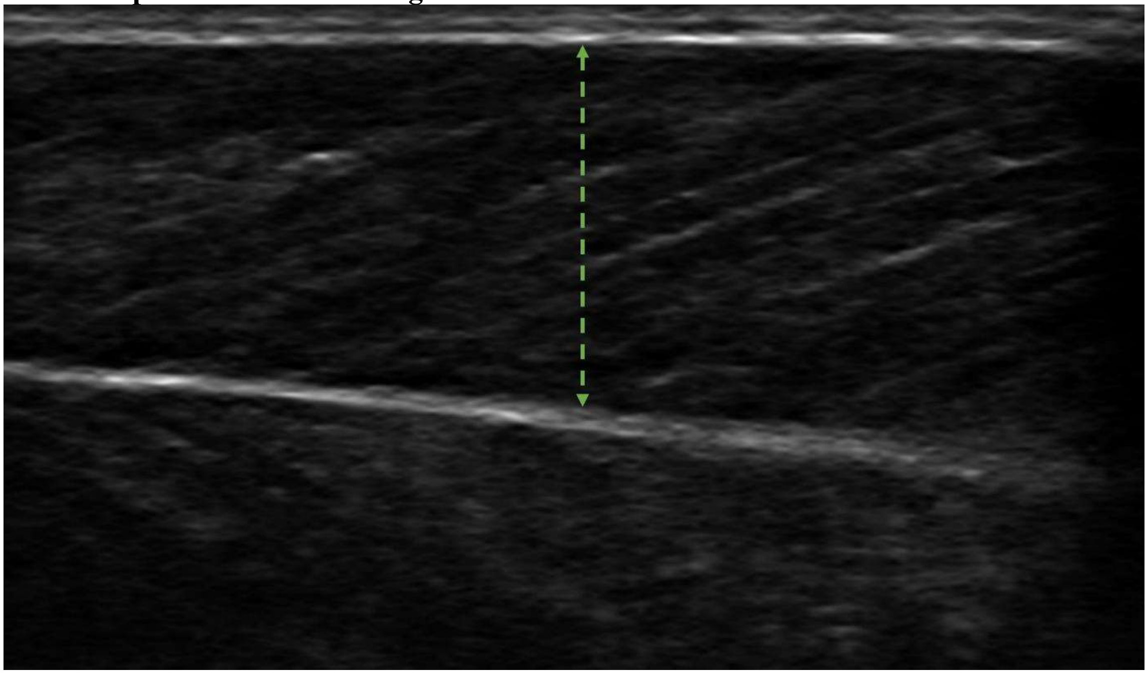

Green arrow denotes the site of measurement from the superficial to the deep aponeurose.

References

Schoenfeld, B. J., Androulakis-Korakakis, P., Coleman, M., Burke, R., & Piñero, A. (2023). SMART-LD: A tool for critically appraising risk of bias and reporting quality in longitudinal resistance training interventions.
